# Evanescent Waves Nuclear Magnetic Resonance

**DOI:** 10.1371/journal.pone.0144483

**Published:** 2016-01-11

**Authors:** El Mohamed Halidi, Eric Nativel, Mohamad Akel, Samir Kenouche, Christophe Coillot, Eric Alibert, Bilal Jabakhanji, Remy Schimpf, Michel Zanca, Paul Stein, Christophe Goze-Bac

**Affiliations:** 1 BioNanoNMRI Laboratoire Charles Coulomb, CNRS University of Montpellier, UMR5221, 34090 Montpellier, France; 2 IES Institut d’Electronique, CNRS University of Montpellier, UMR5214, 34090 Montpellier, France; 3 RS2D 13 rue Vauban 67450 Mundolsheim, France; 4 Institute of Physics, Chemistry and Pharmacy, SDU, Campusvej 55, DK-5230 Odense, Denmark; The Norwegian University of Science and Technology (NTNU), NORWAY

## Abstract

Nuclear Magnetic Resonance spectroscopy and imaging can be classified as inductive techniques working in the near- to far-field regimes. We investigate an alternative capacitive detection with the use of micrometer sized probes positioned at sub wavelength distances of the sample in order to characterize and model evanescent electromagnetic fields originating from NMR phenomenon. We report that in this experimental configuration the available NMR signal is one order of magnitude larger and follows an exponential decay inversely proportional to the size of the emitters. Those investigations open a new road to a better understanding of the evanescent waves component in NMR with the opportunity to perform localized spectroscopy and imaging.

## NMR principles and Evanescent Waves

The principle of Nuclear Magnetic Resonance is based on the detection of the magnetization originating from an ensemble of spins located on the atomic nucleus [1–3]. In living sciences, NMR spectroscopy and imaging of ^1^H, ^13^C, ^23^Na and ^31^P are of great interest and extensively studied [4, 5]. The sample is polarized by a static magnetic field *B*_0_ and spatially encoded by pulse gradients, giving to the nuclear spin distinct precessional movements at well separated Larmor frequencies. Radiofrequency pulses are used to tilt the axis of the magnetization; when the spin system recovers its equilibrium, it generates an electromagnetic field which is classically detected using an inductive coupling in the radiative near-field regime [6]. Radio frequencies are usually emitted from the sample in the range from 1 *MHz* to 1 *GHz*. A good receiving setup can be made generally from a coil wrapped around the sample, a loopless catheter antenna [7] with a tuning/matching circuit [8] or electric potential sensor capacitively coupled to the sample [9]. In this contribution, we propose a completely different approach, with the use of micrometer-sized Evanescent Waves probe (EW-probe) working in a capacitive mode [10] positioned in the vicinity of the object of interest at submillimetric distances, well shorter than the wavelength of the radiated radio-frequency signal. Over the past decades, near-field experiments have been extensively developed and modelized in optics and microwave research in order to gain signal and to overpass the Abbe’s criterion [11]. At least in theory, evanescent waves NMR could be collected also in the non-radiative near-field regime. To our knowledge, evanescent electric fields emitted from nuclear spins have never been explored, even if they potentially contain the same local information conventionally picked-up by coils. Our work presents an alternative method to detect electromagnetic fields which has not been fully exploited in NMR spectroscopy and imaging.

## Methods

### Nuclear Magnetic Resonance setup

**Fig 1:**
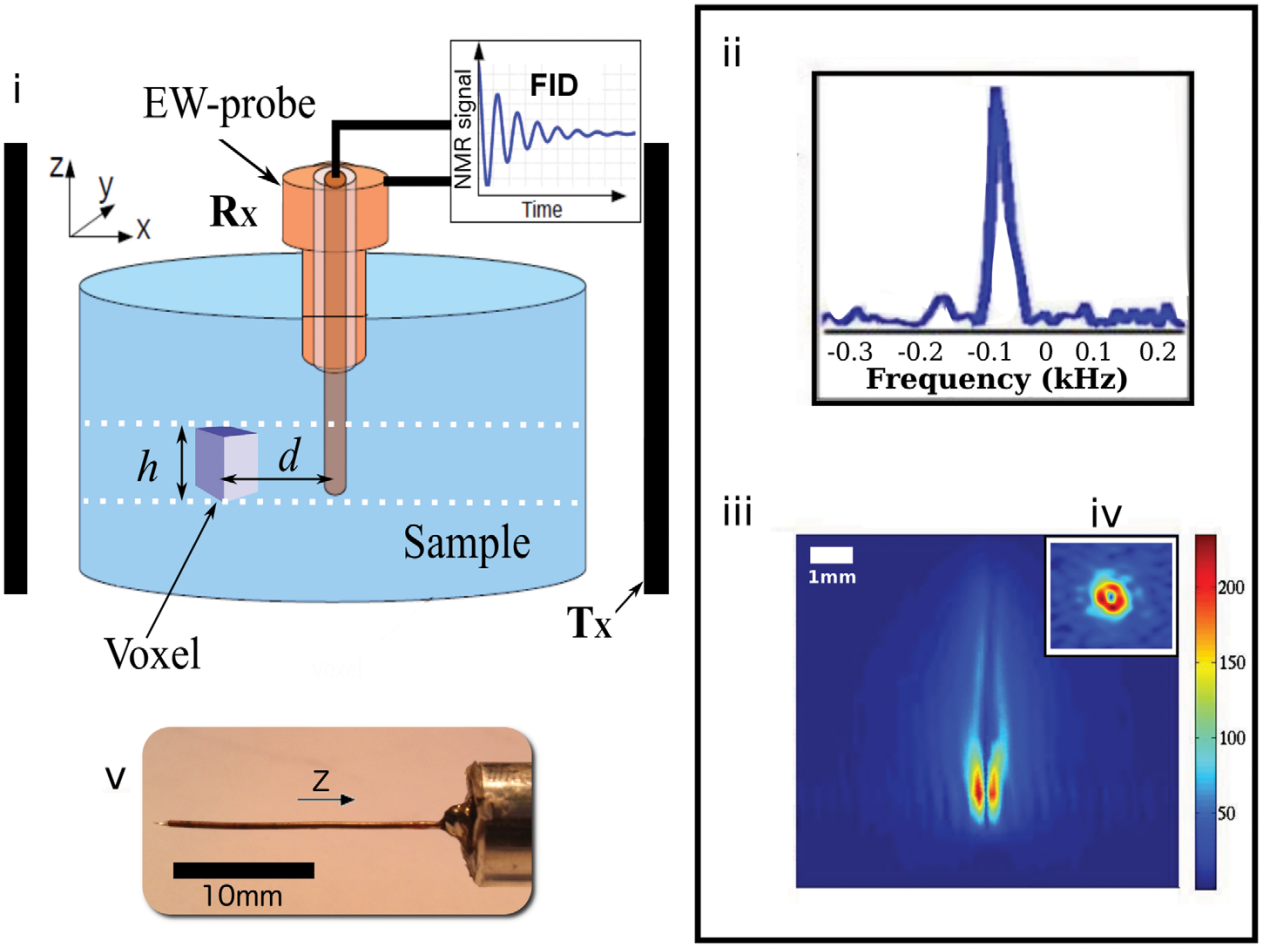
EW-NMR experimental setup. i) shows the transmitter volumic coil Tx, the receiver Rx configuration and the EW-probe in the center of the container filled with a water sample. *h* is the thickness of the selected slice and the height of the parallelipedic voxel. The dashed lines represent a slice selection of thickness *h* with an axial transverse orientation with respect to the magnetic field *B*_0_ oriented along the *Z* axis. *d* is the distance from the center of the voxel to the center of the tip. ii)^1^*H* NMR spectrum collected using the EW-probe configuration. From iii), spatial selection reveals that the NMR signal emitters are located along the tip of the EW-probe and from iv) they are observed on a bright red annulus centered at the tip of the EW-probe. A loss of signal is found on the voxels at the position of the tip itself where there is obviously no emitters. This central point was used as the reference distance (*d* = 0 *μm*) for the computation of the decay profiles of the NMR signal (see Fig 2). v) presents a picture of the EW^*α*^-probe.

^1^*H* NMR and MRI experiments were carried out on a Tecmag Appolo spectrometer at 4.7 *Tesla*, corresponding to a Larmor frequency of 200 *MHz* giving a λ/2*π* ≈ 230 *mm* in air and $\lambda /(2\pi \sqrt{\epsilon_{r}})=26.9\;mm$ in water (*ϵ*_*r*_ = 80). A micro imaging gradient with a maximum strength of 500 *mT*/*m* and a ^1^*H* volumic birdcage coil with a 30 *mm* clear bore were purchased from Resonance Research Incorporate. A Hahn spin echo pulse sequence was used with a repetition time of 3 *s*, an echo delay of 5 *ms* and 80 averages [3]. The matrix data size of the recorded images was 256 × 256 with an in plane *XY* resolution of 128 *μm* × 128 *μm* and a variable slice thickness *h* from 200 *μm* to 800 *μm* with 200 *μm* in step. In the conventional mode, the volumic coil was used as the transmitter *T*_*x*_ and the receiver *R*_*x*_. All the experiments were conducted on a cylindrical container of 22.2 *mm* in diameter filled with 15 *mm* in height of water with the EW-probe positioned in its center (see Fig 1 for the setup).

### Description of the EW-probe

The Evanescent Wave probe is realized from a 50 Ω coaxial cable made from non magnetic copper alloy [10, 12]. The inner conductor is 127 *μm* in diameter and the outer part is 584 *μm*, well below the NMR wavelength. The dielectric material used in this coaxial cable is PTFE with a relative dielectric constant of 2.1. The active part of the probe is the inner conductor pointing out from an extremity of the coaxial cable. Its length can be varied from 0 to 2 *mm*, depending on the desired compromise between sensitivity and spatial resolution. In our study, the influence of the tip geometry has been studied in two cases. The first type of EW-probe, labeled *α*, shows a tip of 2 *mm* in length with a cylindrical shape; the second one, shortened to 500 *μm* and tapered with a conical shape, was named *β*. The coupling of this type of sensor is more efficient with the electric field than with the magnetic one. The sensor acts like a capacitor (capacitive coupling). The situation is quite different from coils that show a better coupling with magnetic field (inductive coupling). In the NMR frequency range, the microprobe acts as an open end circuit and is misadapted with the 50 Ω conventional electronic circuitry. This misadaptation, meaning weak sensitivity, is known to compensate by collecting strong evanescent signal in the vicinity of the sample. On the other hand, its broad band capability gives the opportunity to detect different nuclear spin species without any modification of the EW-probe and of the applied magnetic field.

### The EW-NMR experimental setup

Fig 1i) presents a sketch of a typical configuration for the Evanescent Wave Nuclear Magnetic Resonance apparatus. From the conventional experimental configuration, the receiver *R*_*x*_ is disconnected from the volumic coil and connected directly to the EW-probe. A 30 *dB* low noise (Noise Factor: 1.2) preamplifier from Miteq is used before the digitization by the analog to digital converter of the spectrometer. The axis of the EW-probe is aligned with the static magnetic field *B*_0_ along the *Z* axis in order to capture the NMR signal emitted from a *XY*-slice of thickness *h* selected using pulsed field gradients. Each voxel can be seen as an emitter characterized by *d*, its distance to the tip of the EW-probe and by its height *h*. Hence *XY*-image can be analyzed in terms of a series of identical emitters (supposing the sample is homogeneous) distributed in a disk centered at the tip of the EW-probe.

Fig 1ii) presents the ^1^*H* NMR spectrum recorded when no spatial selection is applied *i.e.* when the signal from the whole sample is collected. A single peak is observed with a full half width at half maximum of about 50 *Hz*, as expected from the homogeneity of the static magnetic field *B*_0_. *T*_1_ and *T*_2_ relaxation experiments have been performed using standard pulse echoes sequences. The corresponding relaxation rates were found in good agreement with the ones measured from the conventional detection configuration (data not shown here). It should be noticed that no signal is collected when the EW-probe is too far from the sample, for example at a distance ≈ 1 *mm* above the surface.

Fig 1iii) and 1iv) show sagittal and axial selected images crossing the tip of the EW-probe, respectively. The selections are performed with the help of classical pulsed field gradients orthogonal to the corresponding orientations. In this case, the NMR signal emitters can be spatially discriminated and a clearly hot spot is observed in the center of the images. Interestingly, this unusual strong NMR signal is localized in the vicinity of the tip of the EW-probe with a rapid decay giving almost zero signal for distances larger than 1 *mm*. A clear loss of signal is observed in the center of the hot spot, corresponding to the physical tip of the EW-probe where there is obviously no sample.

## Results

**Fig 2:**
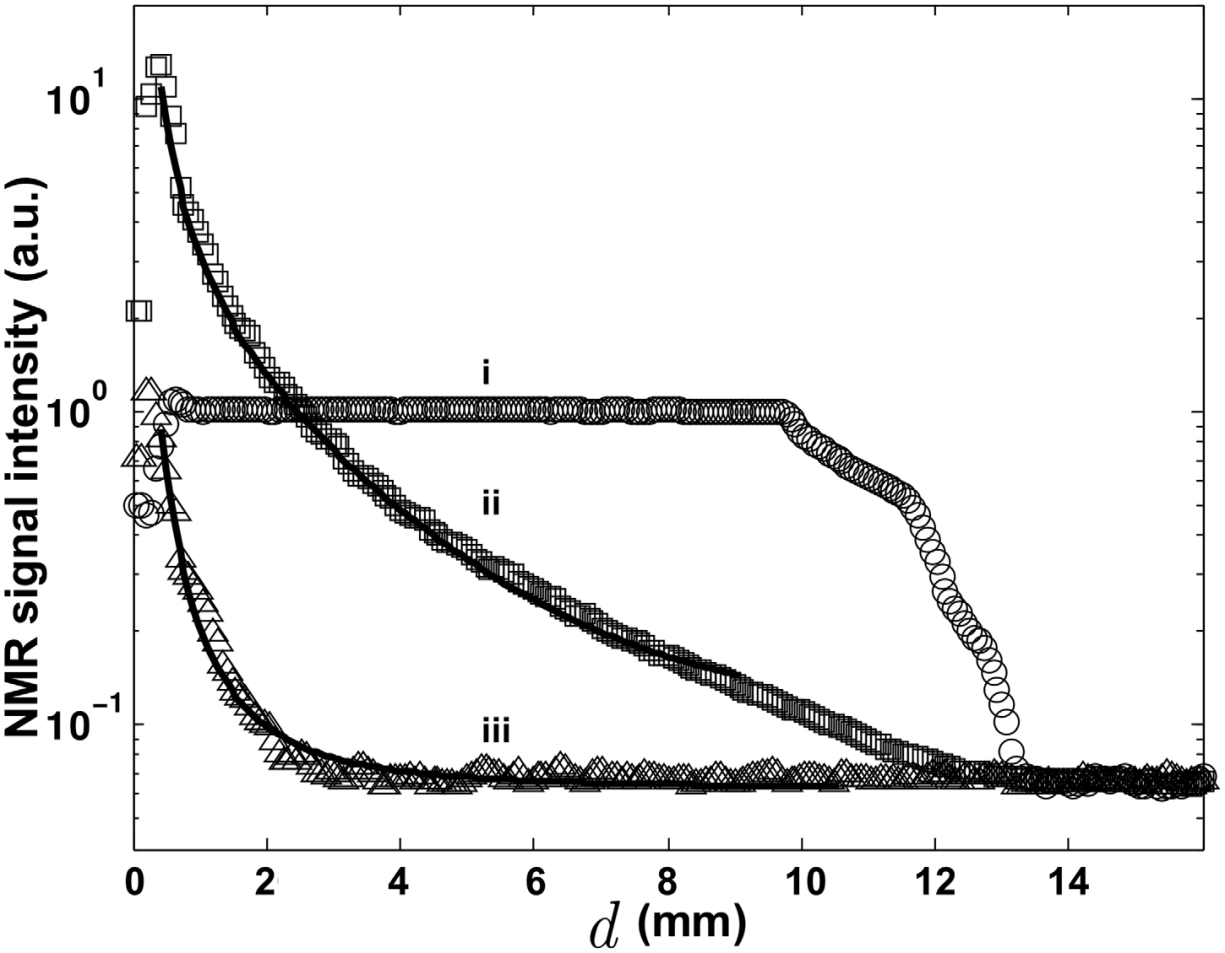
NMR signal intensity decays with respect to the distance to the tip of the EW-probe. Receiver Rx is connected to: i) 30 *mm* birdcage volumic coil, ii) cylindrical EW^*α*^-probe and iii) conical EW^*β*^-probe. In the three experiments, there is almost no detected signal on the tip of the EW-probe (*d* = 0 *μm*) since there is no emitter.

Fig 2 presents the comparison of the decay profiles of the two investigated EW-probes, labeled *α* and *β*.

**Fig 3:**
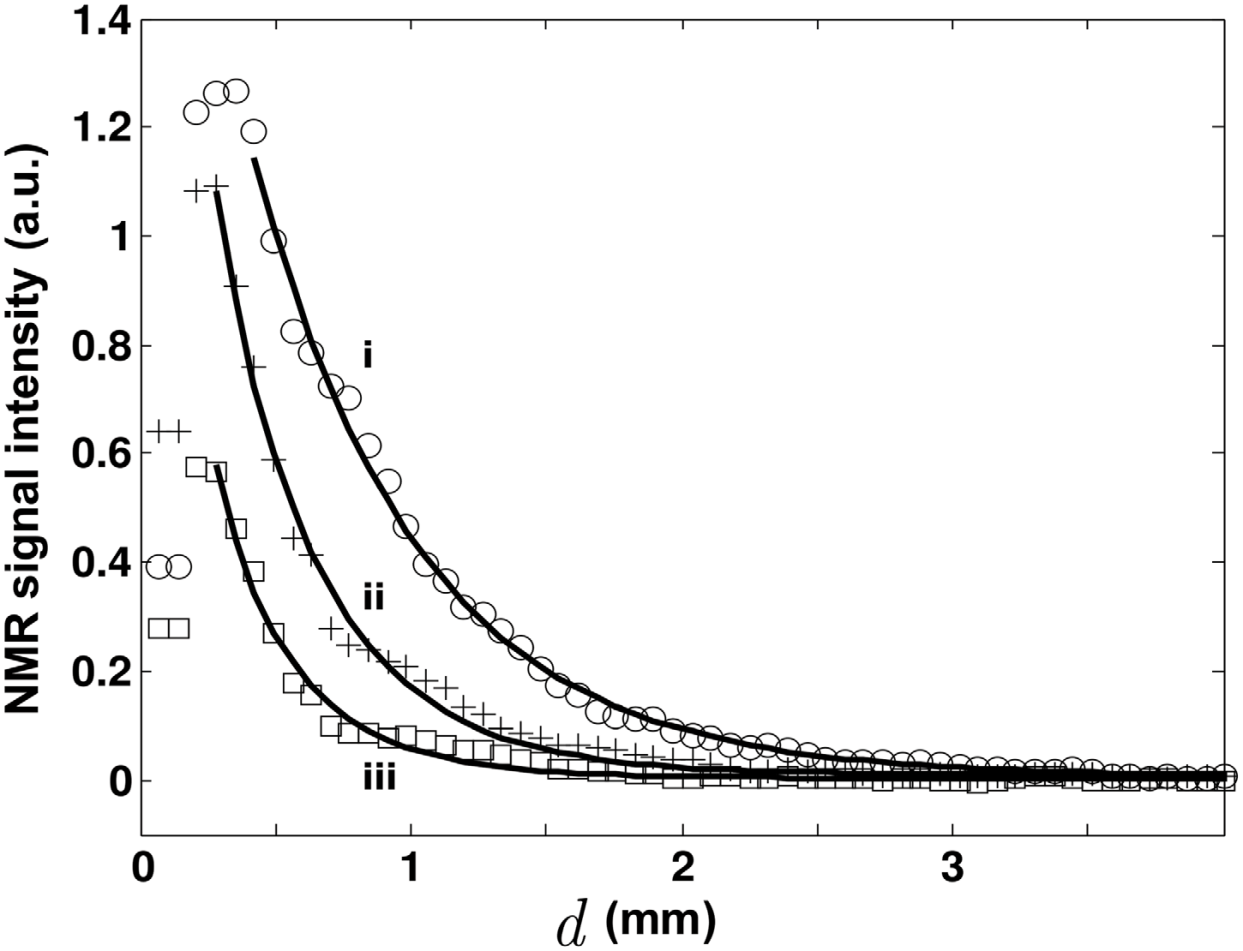
NMR signal intensity decays with respect to the distance to the tip for the conic EW^*β*^-probe for various height *h* of the emitters, corresponding to i) 600 *μm*, ii) 400 *μm* and iii) 200 *μm*. For the clarity of the figure, only three experiments are presented here.

These measurements are referenced to a volumic birdcage coil with a 30 *mm* clear bore that shows an almost flat-top profile till the end of the cylindrical container. The decay profiles of the EW-probes are radically different from the one measured with the volumic coil. At a distance of about 300 *μm* from the center of the tip, a maximum of signal is reached and a rapid decay is observed with the distance *d*. The loss of signal in the center of the hot spot can be attributed to the presence of the tip and the generation of eddy-currents in the conductive material. This small electric effect that affects the images over two voxels on the tip is also observed with the volumic coil. This artifact becomes rapidly negligible at distance larger than 300 *μm* (see Fig 2i) for the volumic coil) suggesting that the exponential decays presented in Figs 2 and 3 is due to another physical phenomenon.

Some differences are also observed in the collected signal intensities between *α* and *β* EW-probes. If the EW^*α*^-probe collects one order of magnitude more signal than the EW^*β*^-probe, the localization is improved in the *β* case. As expected, the geometry of the EW-probe controls the spatial selectivity and defines the potential exploration of the sample.

We turn now to the study of the exponential decay with variable height *h* of the emitters. The EW^*β*^-probe has been selected for this study since the localization of the emitters is more pronounced than the EW^*α*^-probe. *h* has been varied from 200 *μm* to 800 *μm* by decreasing the amplitude of the selection pulse field gradient. Fig 3 presents the different decay profiles for the EW^*β*^-probe. By increasing the height of the volume containing the emitters, more NMR signal is collected together with a change in the decay profile. The total intensity of the signal increases with respect to *h* and saturates as soon as the slice thickness *h* is larger than the length of the tip (data not shown here). In the following section, we discuss these observations in terms of the detection of evanescent waves originating from the relaxation of the nuclear magnetization.

## Discussion

**Fig 4:**
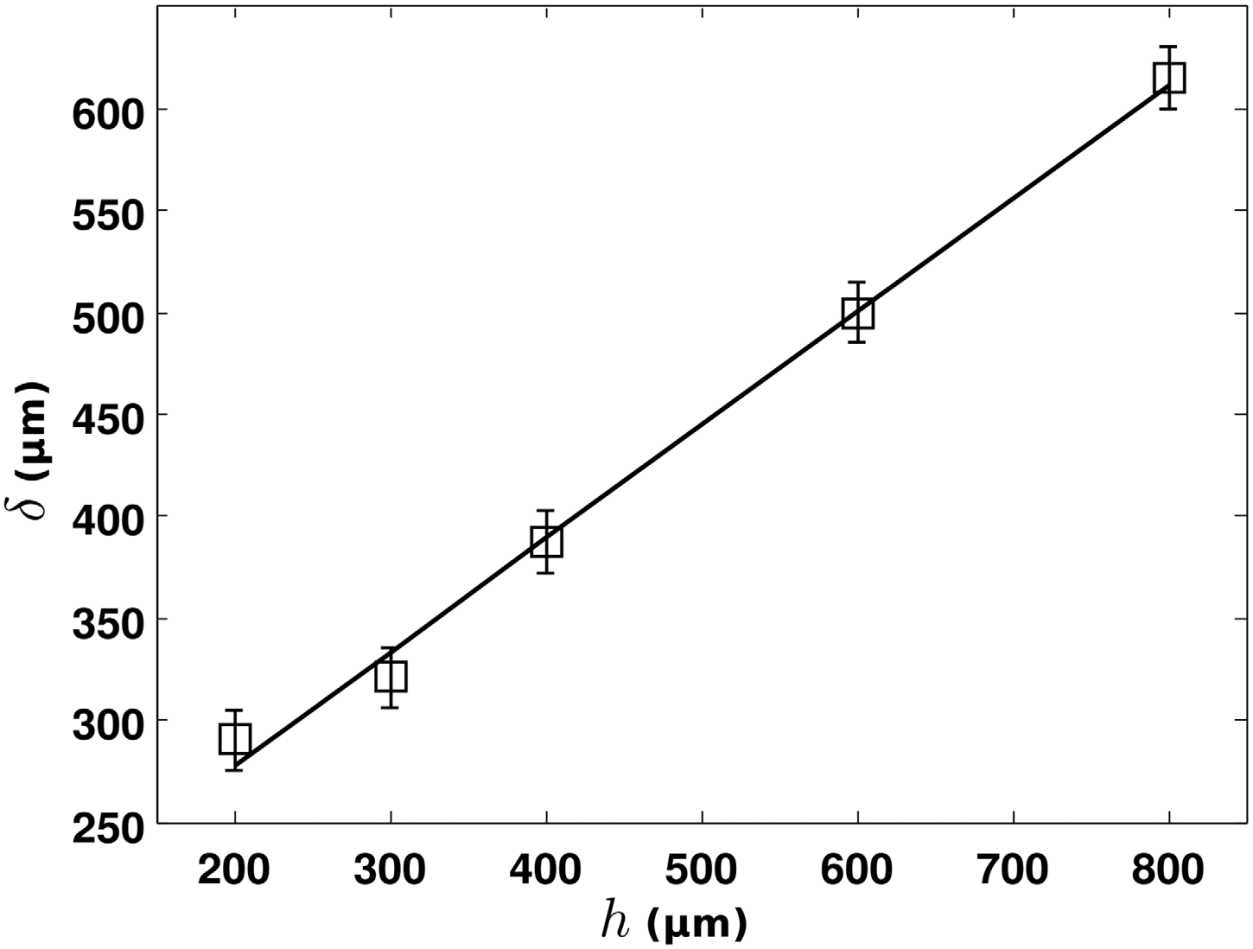
Fitting parameter *δ* as a function of the emitters height *h*.

Our approach of the detection of the nuclear spins signal combines two ingredients. Firstly, we investigate the electric field component associated to the time dependence of the magnetic field ($\nabla \times \vec{E}=-\partial \vec{B}/\partial t$) of the free induction decay signal, *i.e.* looking for a capacitive coupling between the sensor and the magnetization from the nuclear spins of the sample. Secondly, the near-field component (Ψ_*NF*_) of the electric field is collected using an EW-probe at distances well shorter than λ/2*π*. This setup gives the opportunity to detect the evanescent waves component (Ψ_*EW*_) which contributes significantly to the total electromagnetic field and dominates the propagative near-field component (Ψ_*PW*_), which is usually the unique component measured from coils wrapped around the sample, and the far-field (Ψ_*FF*_) exploited only recently by collecting “traveling-waves” [13]. In such an experimental configuration, we explore the possibility to use the micro-sized tip of the EW-probe to transform the evanescent waves (Ψ_*EW*_) from the sample into propagative waves and to transmit them to the receiver Rx connected directly to a 30 *dB* preamplifier and the ADC converter of the spectrometer (see Figs 1 and 2). If this is the case, an interesting feature is predicted from Near Field theory that the exponential decay parameter *δ* is related to the size of the emitters *h* [11, 14]. Indeed, *δ* and *h* are expected to be proportional and that behavior is the purpose of the study presented in the Figs 3 and 4. Hence, the experimental data measured using the *α* and *β* EW-probes were tentatively fitted by the following equation:

$$\Psi =\overset{\Psi_{NF}}{\overset{︷}{\underset{\Psi_{EW}}{\underset{︸}{\Phi \exp(-\Delta /\delta )}}+\underset{\Psi_{PW}}{\underset{︸}{\Lambda /\Delta^{2}}}}}+\Psi_{FF}$$ (1)

where Ψ_*NF*_ is the near-field component, Φ is the amplitude of the evanescent waves component Ψ_*EW*_, Δ = *d* − *d*_0_ the distance from the surface of the tip to the center of a given voxel, *d*_0_ = 63.5 *μm* is the radius of the tip of the EW-probe, Λ is the amplitude of the propagative near-field component Ψ_*PW*_ and Ψ_*FF*_ represents the far-field component that can be approximated by a constant in the range of the investigated distances. Fig 2 shows the results for two types of EW-probe and their best fits according to Eq 1. As expected from considering the different size and geometry of the tips, more NMR signal is collected with the EW^*α*^-probe than from the shorter one EW^*β*^-probe, giving $\bar{\Psi_{NF}^{\alpha }}\approx 10\;\bar{\Psi_{NF}^{\beta }}$. The localization of the signal is clearly enhanced in the case of the EW^*β*^-probe which presents a tapered active part. Indeed, the ratio between the exponential decay parameters is found to be *δ*_*α*_ ≈ 60 *δ*_*β*_, and integrating the different contributions, one obtains $\bar{\Psi_{EW}^{\alpha }}\approx \bar{\Psi_{PW}^{\alpha }}$ and $\bar{\Psi_{EW}^{\beta }}\gg \bar{\Psi_{PW}^{\beta }}$ for the *α* and *β* EW-probe, respectively. The tapered EW^*β*^-probe is clearly found to collect mainly evanescent waves in the close vicinity of the tip, suggesting it is well adapted to perform localized spectroscopy.

We turn now to explore the dependence of *δ* with *h*, the size of the volume containing the emitters, in order to confirm the observation of evanescent NMR waves. In the following, we will concentrate on the EW^*β*^-probe and the label *β* will henceforth be omitted. The fits of the decay profiles are presented in Fig 3 and the trend for *δ* as a function of *h* is shown in Fig 4. For the clarity of Fig 3, only three measurements are presented, for *h* = 600*μm*, 400*μm* and 200*μm*. As expected, a decrease of the collected signal intensity can be noticed when reducing the size of the emitters *h*. From the fits, we can estimate that, in the recorded NMR signal, the propagative near-field component Ψ_*PW*_ is not contributing significantly while the non propagative evanescent waves component Ψ_*EW*_ part is dominating. In Fig 4, the exponential decay parameter *δ* as a function of *h* and its linear fit are presented.

In the Near Field theory [11, 14], one expects a linear relation between h and *δ*. In our experiment we found a ratio Δ*h*/Δ*δ* = 1.8±0.2. Finally, we want to point out, from the decay profile in Fig 3iii), that we can obtain an extreme localization of the NMR signal, with more than 90% of the signal intensity detected at distance shorter than 600 *μm*, corresponding to the approximate length of the tip. This means that NMR spectroscopy could be performed in the corresponding volume without using any pulse field gradient. Further work is under progress to change the tip geometry in order to enhance the localization of the collected region and to perform imaging using micro-positioning. The expected features for the detection of evanescent NMR waves and their conversion to propagative ones are definitively demonstrated. They have been successfully collected to perform spectroscopy and imaging in a way that could revolutionize the conventional NMR approach.

## Conclusion

A novel way to detect NMR signals is demonstrated by using electric field EW-probes exhibiting a capacitive coupling with the nuclear spins magnetization originating from the sample. In our method, it is possible to approach up to contact the sample where the evanescent waves NMR signal in the non radiative regime is expected to be about one or two orders of magnitude more intense than for the propagative near-field and far-field components. In agreement with near-field principles, our NMR study demonstrates the relation giving an exponential decay of the signal intensity with the position and size of the emitters with respect to the tip of the EW-probe. NMR is demonstrated to be a powerful technique with potential applications in the propagative near-field regime by using evanescent electric field waves to perform spectroscopy or imaging.
